# Population-based concordance of *Chlamydia trachomatis* genotypes between ocular and urogenital samples in Nauru

**DOI:** 10.1128/spectrum.03205-24

**Published:** 2025-10-08

**Authors:** Kym Lowry, Sue-Chen Apadinuwe, Mitchell Starr, Susan Star, Kathleen D. Lynch, Anthony W. Solomon, Philip Cunningham, Stephen Lambert, David Whiley, John Kaldor, Susana Vaz Nery

**Affiliations:** 1 The University of Queensland Centre for Clinical Research (UQCCR), The University of Queensland 1974, Brisbane, Queensland, Australia; 2 Ministry of Health and Medical Services, Denig, Republic of Nauru; 3 NSW State Reference Laboratory for HIV, St Vincent’s Centre for Applied Medical Research, St Vincent’s Hospital, Sydney, Australia; 4 Global Neglected Tropical Diseases Programme, World Health Organization 3489, Geneva, Switzerland; 5 National Centre for Immunisation Research and Surveillance, Westmead, New South Wales, Australia; 6 The Kirby Institute, University of New South Wales 7800, Sydney, Australia; ARUP Laboratories, Salt Lake City, Utah, USA

**Keywords:** *Chlamydia trachomatis*, trachoma, urogenital, genotyping, Nauru

## Abstract

*Chlamydia trachomatis* causes sexually transmitted urogenital infections, as well as ocular infections, the latter mostly spread between children and responsible for the blinding disease trachoma. The strains causing these two types of infection are generally distinct, but there is some evidence that urogenital strains can infect the eye and cause conjunctivitis, possibly indicating transmission on fingers of adults or adolescents to children. In what we believe is the first study of its kind in a setting of high prevalence of both urogenital and ocular infection, we characterized *C. trachomatis* strains from 107 ocular and 95 urine samples collected through cross-sectional population surveys in Nauru, employing household-based sampling. Ocular samples were collected during a national baseline prevalence survey in 2019 in children aged 1–9 years. Urine samples were collected from adults participating in a national survey before (March 2020) and after (December 2020) a national mass drug administration (MDA) of azithromycin for trachoma in April 2020. We used a cluster survey design to recruit participants, with clusters based on Nauru’s districts and a predetermined number of households randomly selected in each cluster. There was no attempt to recruit from the same households across the three survey rounds. Only genotype C, recognized as an ocular strain, was identified in eye specimens from children, whereas six urogenital genotypes (D, E, G, J, Ja, and L1) were detected in adult urine samples, with genotype D most frequent. We, therefore, concluded that, in this highly endemic setting for both urogenital and ocular infection with *C. trachomatis,* there were two distinct networks of transmission with no evidence of crossover, at least at the population level.

**IMPORTANCE:**

Chlamydia infections are a public health issue with two broad manifestations: ocular infections, mostly found in children, and sexually transmitted infections of the genital tract and anus that can lead to adverse reproductive health outcomes. While generally caused by different *C. trachomatis* strains, there is some evidence that strains considered to be predominantly sexually transmitted can infect the conjunctiva and cause signs resembling trachoma. Possible strain crossover has raised concern about whether eye infection with genital *C. trachomatis* strains confers a drive toward visual impairment and blindness, and the potential for such infections to lead to overestimates of trachoma prevalence. In what we believe to be the first study of its kind, we identified distinct ocular strains in pediatric ocular swabs and urogenital strains in adult urine specimens in Nauru and concluded that urogenital *C. trachomatis* strains are not contributing to ocular disease despite the high prevalence of urogenital chlamydia.

## INTRODUCTION

*Chlamydia trachomatis* is a bacterium that infects ocular, urogenital, and anal epithelia. Trachoma is caused by an inflammatory response in the conjunctival tissue of the eyelid to ocular infection with *C. trachomatis*. In April 2024, an estimated 103 million people lived in areas considered to have a public health problem from trachoma (1), with young children most likely to have infection and the early clinical manifestation known as trachomatous inflammation—follicular (TF) (2). Repeated infections can lead to long-term inflammation, with resulting scarring of the eyelid causing inversion of the eyelashes. These can damage the cornea and, potentially, lead to blindness (3, 4). *C. trachomatis* of the urethra, cervix, or anus is a common sexually transmitted infection associated with local inflammation at the infection site (5), which adversely affects reproductive health outcomes and enhances the risk of HIV transmission. In 2020, there were an estimated 128.5 million new sexually transmitted *C. trachomatis* infections globally among 15–49-year-olds (6). Some strains of *C. trachomatis* can spread from the genital epithelium to monocytes of the lymphatic system, causing the disease known as lymphogranuloma venereum (LGV) (7).

*C. trachomatis* comprises two biovars according to anatomical localization at the tissue level; the trachoma biovar involves epithelial infections and includes ocular and urogenital strains, while the lymphogranuloma venereum biovars pass systemically through the lymphatic system (8). Genotyping of *C. trachomatis* has led to strain classification into 19 genotypes, as defined by sequence variation in the outer membrane protein, encoded by the *ompA* gene (9, 10). Those associated with ocular infection and trachoma are *ompA* genotypes A, B, Ba, and C (11); genotypes D, Da, E, F, G, Ga, H, I, Ia, J, and K are generally associated with urogenital *C. trachomatis* infections commonly known simply as chlamydia, while genotypes L1, L2, and L3 are associated with LGV (10, 12, 13). *C. trachomatis* infections caused by genotypes D–K can also occur in the eye and lead to a condition that is clinically indistinguishable from the TF caused by types A-C, usually in people with concurrent urogenital infections, their sexual partners, or household contacts (14, 15).

Nauru is a small coral island located in the central Pacific. In the most recently published 2021 Nauru Population & Housing Census, the total population of Nauru was 11,680 across 2,021 households (average of 5.8 people per household) with a median age of 21.6 years and life expectancy from birth 63.9 years (16, 17). The negative net migration rate (−1.0%) from 2011 to 2021 is explained by repatriation of I-Kiribati and Tuvaluan workers, but data on migration traffic, particularly arrivals and departures involving surrounding island nations such as Republic of Kiribati and Solomon Islands, cannot be determined as these data are not collected by the Nauruan Customs and Immigration Office (16). In addition, there are offshore Australian immigration detention facilities holding asylum seekers who are not counted in the Nauruan population.

Nauru has a high prevalence of both ocular and urogenital chlamydial infections. A nationwide baseline trachoma prevalence survey in July 2019 estimated, among 1-9-year-olds, a TF prevalence of 22% and over 30% prevalence of both *C. trachomatis* in eye swabs and anti-*C. trachomatis* antibodies in capillary blood (18). The proportion of routinely collected urogenital specimens positive for chlamydia infection steadily increased from 3.4% to 13.6% between 2011 and 2015 (19). A national household survey was conducted in 2020 to estimate the prevalence of urogenital chlamydia and gonorrhea in adults aged 18–29 years through the collection and analysis of urine specimens immediately prior to a round of mass drug administration (MDA) of azithromycin for trachoma, and the survey was repeated 8 months after MDA (manuscript in preparation). Urogenital chlamydial infections have been recorded at high prevalence in a number of Pacific Island countries (20), while ocular chlamydial infections have been detected at high levels in Kiribati, with much lower prevalences in other Pacific island countries (21).

The published literature on the role of urogenital strains of *C. trachomatis* on trachoma-like eye disease has so far been limited to case reports and case series. There are concerns that in settings where there is a high burden of both trachoma and urogenital chlamydial infections, there may be crossover of strains (22) and that the presence of endemic urogenital infection may contribute to ocular infections in children and increasing the risk of vision loss (22–24), or (even if urogenital strains are not increasing the risk of long-term trachoma sequelae) complicating monitoring and evaluation of efforts to eliminate trachoma. We therefore undertook what we believe to be the first population investigation of a crossover between ocular and urogenital *C. trachomatis* strains in a high-prevalence region.

## MATERIALS AND METHODS

### Sample collection

#### Ocular specimens

In the context of a national trachoma survey in July 2019 (18), conjunctival swabs were collected from participating children aged 1–9 years to test for the presence of *C. trachomatis*. Briefly, for the trachoma component, 20 clusters with approximately equal population sizes of 560 inhabitants were created using 15 administrative areas in Nauru (14 districts and one settlement), plus five further subdivisions drawn from the three most populated areas. Approximately 23 households (from an average 90 per cluster) were randomly sampled from each cluster using a pre-existing household list, with individual eligibility restricted to Nauruan citizens. A total of 277/780 *C*. *trachomatis* positive swabs, each taken from an individual child, were detected as previously reported (18) and then stored at −20°C.

#### Urine specimens

Cross-sectional surveys were undertaken one week before (March–April 2020) and 8 months after (December 2020) the azithromycin MDA conducted in April 2020, with the same methods used for both rounds. For the urogenital survey component, we targeted Nauruan adults aged ≥18 to ≤ 29 years. Both the baseline and follow-up surveys used a random sampling approach using the same 20 clusters as those used for the trachoma survey implemented in July 2019. For each of the two surveys, half of the clusters were randomly selected for involvement in the study. In each cluster, half of the existing households were sampled and randomly selected from a pre-existing list of households. Participants were provided a sterile urine pot for their first pass urine (FPU) sample and asked to provide a 1050 mL sample. Research teams carried specimens in the field for up to 8 hours. Each specimen was aliquoted into a COBAS (Roche Diagnostics, Australia) media tube at the end of the day in preparation for transport to Australia at room temperature.

To calculate the probability of the same household being sampled across both surveys, the percentage of households sampled in each survey was determined in each cluster and multiplied together, followed by a final average calculation across the clusters (data not shown).

### *C. trachomatis* detection

Ocular swabs and urine were initially screened for *C. trachomatis* using the cobas CT/NG test (Roche Diagnostics, Australia) at St Vincent’s Center for Applied Medical Research, Sydney, Australia (18). Of the urine specimens collected, 80/369 (21.7%) in the pre-MDA survey and 49/345 (14.2%) in the post-MDA survey tested positive for *C. trachomatis,* and these 129 samples were stored at −20°C until required for this genotyping study.

De-identified specimens positive for *C. trachomatis* were shipped frozen to the UQCCR laboratory in Brisbane for subsequent genetic characterization. We extracted nucleic acid from a 200 µL aliquot of each urine or ocular swab sample using the MagNA Pure 96 System (Roche Diagnostics, New South Wales, Australia) into a final 50 µL elution. Polymerase chain reaction and Sanger sequencing were performed using this extracted nucleic acid.

### Genotyping: *C. trachomatis* by anatomical site

The Giffard et al*.* (2018) method was adapted to a Sanger sequencing approach to perform *C. trachomatis* genotyping directly on all sample extracts. We initially targeted ofr, an amplified region covering a fragment of the major outer membrane protein encoded by *ompA* for all samples, and 33 samples for additional rg1, a fragment from the hypothetical gene defined by Jali-1891 in the *C. trachomatis* B_Jali20 genome (25). Where possible, we selected at least one sample representing each *ompA* genotype for further characterization using rg1. Samples were generally selected across a range of Ct values so as not to bias high bacterial load samples only. The rg1 region of interest contains two SNPs that define four haplotypes that align with greater *C. trachomatis* genome phylogeny. Sequencing *ompA* and rg1 fragments provided high combinatorial resolving power to i) discriminate between ocular and urogenital lineages, ii) discriminate classical ocular lineages and three atypical Australian ocular lineages with urogenital-type genome backbones, and iii) discriminate the entire species into their major evolutionary lineages, all to confirm anatomical site-specific genotyping (25). A 20 µL reaction mix consisted of 10 µL QuantiTect SYBR Green PCR mix (Qiagen), 0.45 µM each primer (ofr-F 5′ TCCTACTGCAATACCGCAAG 3′ and ofr-R 5′ TGAACCAAGCCTTATGATCG 3′, or rg1-F 5′ CCC ATT GCC GAG AGA TAA AA 3′ and rg1-R 5′ CTC CTG CGG AGG TTA GAT TG 3′), and 5.0 µL specimen extract. Cycling conditions consisted of 95°C for 15 min for enzyme activation and 45 cycles of 94°C for 15 sec and 60°C for 20 sec and 72°C for 60 sec; cycling was performed on a Rotor-Gene Q instrument (Qiagen). Amplification of ofr and rg1 regions was determined by high-resolution melting, which is more sensitive than gel electrophoresis in detecting amplicon. A selection of samples representing most *ompA* genotypes in this study (C, D, E, G, J, and Ja) were further sequenced with the rg1 target for confirmation of genotyping resolution. A single ocular swab, typed as L1, could not be amplified using the rg1 assay. Expected amplicon sizes for ofr (*ompA*) and rg1 were 1,015 bp and 354 bp, respectively, and were Sanger-sequenced, dual direction (forward and reverse) by service providers, Australian Genome Research Facility (Brisbane, Australia).

### Phylogenetic analysis

To illustrate the genetic relationship of *C. trachomatis* detections with the Nauruan population, we constructed an unrooted phylogenetic tree. Completed *C. trachomatis* ofr sequences were aligned using ClustalW in BioEdit Sequence Alignment Editor (version 7.0.5.3) (26). The phylogenetic tree was generated using Geneious Prime (version 2019.2.3) with the maximum likelihood algorithm and Tamura Nei model (27). The resulting tree was visualized and annotated using iTOL (28).

## RESULTS

### Ocular specimens

In the initial trachoma survey, 272/780 (34.9%) participant swabs were PCR-positive for *C. trachomatis* (Table 1) (18). Five extra swabs PCR-positive for *C. trachomatis* were dispatched for genotyping despite being unmatched, due to labeling errors. Genotyping using the main characterizing *ompA* target was successful for 107/277 (38.6%) of the ocular swabs (Table 1), with original C_T_ values for *C. trachomatis* detection ranging from 19.75 to 39.56. The 1015 bp amplicon was almost sequenced in its entirety in both directions, for both ocular swabs and urine samples, with approximately a 900 bp sequence obtained in each direction. On the basis of *ompA* (ofr target) sequencing, all ocular chlamydial specimens were genotype C (107/107; 100%) (Table 2). Most samples, 92/107 (86.0%), shared 100% sequence identity, while single-base pair variations were detected in 15 samples. For confirmatory genotyping resolution, 15 ocular samples were further sequenced at the rg1 target, which is used also to confirm ocular strains (GC) as belonging to the classic C cluster in T2 lineage within the trachoma biovar, according to Giffard et al*.*’s proposed typing scheme (25) (Table 3).

**TABLE 1:** Prevalence and genotyping rates of *C. trachomatis* in urine and ocular specimens

Sample type	*C. trachomatis* PCR-positive (%)	*ompA* gene (ofr fragment) - sequenced (%)	rg1 fragment - sequenced^*a*^
Ocular swab^*b*^	272/780 (34.9)	107/277 (38.6)	15
Urine (pre-MDA)	80/369 (21.7)	54/80 (67.5)	16
Urine (post-MDA)	49/345 (14.2)	41/49 (83.7)	2
Urine (total)	129/714 (18.1)	95/129 (73.6)	18

^*a*^ rg1 sequencing was performed for selected samples representing different ofr genotypes.

^*b*^ Ref (18).

**TABLE 2:** *C. trachomatis* genotypes in urine and ocular specimens

Sample type	Genotype (ofr)							Total
	C	D	E	G	J	Ja	L1	
Ocular swab	107 (100%)	–^*a*^	–	–	–	–	–	107
Urine (pre-MDA)	–	21 (38.9%)	13 (24.1%)	4 (7.4%)	3 (5.6%)	13 (24.1%)	–	54
Urine (post-MDA)	–	14 (34.1%)	17 (41.5%)	3 (7.3%)	3 (7.3%)	3 (7.3%)	1 (2.4%)	41
Urine (total)	–	35 (36.8%)	30 (31.6%)	7 (7.4%)	6 (6.3%)	16 (16.8%)	1 (1.1%)	95

^*a*^ “–” indicates not applicable.

**TABLE 3:** Dual *C. trachomatis* genotyping targets ofr (*ompA* gene) and rg1 in urine and ocular specimens

Sample type	*n*	Genotyping alleles		Description	Lineage
		ofr allele	rg1 haplotype		
Urine (pre-MDA)	5	D	GA	D in T2	T2
Urine (pre-MDA)	3	E	GT	Typical E	T1
Urine (pre-MDA)	1	G	GA	Typical G	T2
Urine (pre-MDA)	2	J	GA	Non-ocular T2 not D or G	T2
Urine (post-MDA)	2	J	GA	Non-ocular T2 not D or G	T2
Urine (pre-MDA)	2	Ja	GA	Non-ocular T2 not D or G	T2
Urine (pre-MDA)	3	Ja	GT	Ocular AusC	T1
Ocular swab	15	C	GC	Ocular classic C	Ocular

### Urine specimens

Sanger sequencing of the ofr (*ompA* gene) target was successful for 54/80 (67.5%) pre-MDA and 41/49 (83.7%) post-MDA urine specimens (Table 1). Original cobas CT/NG assay C_T_ values for positive *C. trachomatis* detection ranged from 23.09 to 34.05. Urine specimens yielded *C. trachomatis* genotypes D, E, G, J, and Ja, apart from one which was L1 (Table 2). Genotype D was the most prevalent (35, 36.8%), followed by E (30, 31.6%), Ja (16, 16.8%), G (7, 7.4%), and J (6, 6.3%). A similar pattern of genotypes was detected before and after MDA. Eighteen urine samples, that represented most *ompA* genotypes by the ofr target, were further sequenced for the rg1 target. Combining ofr and rg1 loci identified major different phylogenetic lineages associated with common and rarer UGT genotypes (GA, GT) in sampled urine specimens (Table 3) (25).

**Fig 1:**
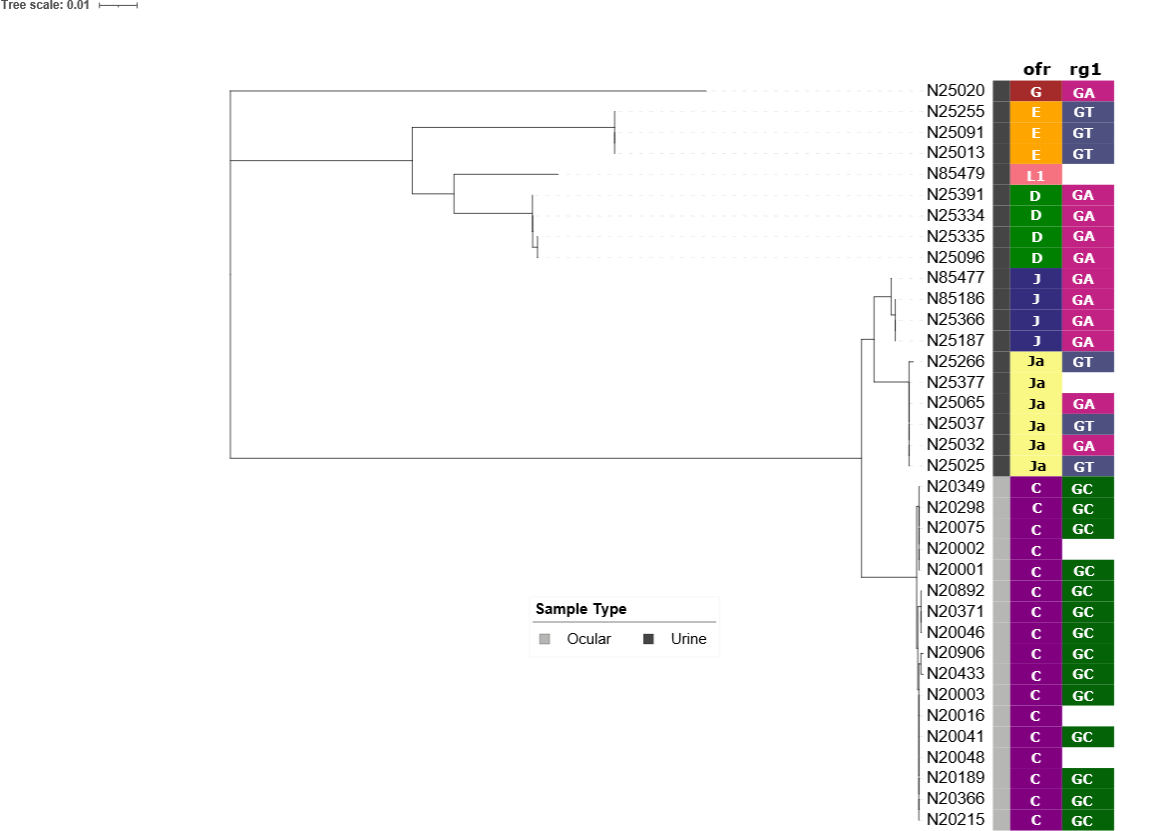
Relationship between the anatomical site of collection and *C. trachomatis* phylogeny. The phylogenetic tree is based on ofr sequences, and *ompA* alleles (ofr and rg1) are indicated with colors.

Representative samples for each ofr genotype and their paired rg1 group were included in the phylogeny and revealed distinct grouping according to the sample type (urine and ocular) for both loci (Fig. 1).

For the samples that we could not type using the Sanger sequencing method, the original cobas CT/NG assay C_T_ values for *C. trachomatis* detection ranged from 23.07 to 41.46 for urine samples (34/129, 26.4%) and 22.7–43.07 for ocular samples (170/277, 61.7%).

## DISCUSSION

In Nauru, we completed what we understand to be the first study to simultaneously attempt genetic characterization of ocular and urogenital specimens at the population level in the same endemic setting. Our goal was to investigate crossover of types at a population, rather than individual level. Even in endemic settings, it would be rare to find *C. trachomatis* infection at both urogenital and ocular sites in the same individual as it is well established that urogenital infection is sexually transmitted among older adolescents and adults, whereas ocular infection is a phenomenon found among children spread through nasal and ocular discharge among peers. Furthermore, there was no attempt to link children and adults across surveys as the relevant transmission events may have happened elsewhere and in the past. We found that eye infections in children were attributable to a single serovar of trachoma-related *C. trachomatis* (genotype C), while six different urogenital genotypes, D, E, G, J, Ja, and L1, were detected in adult urine specimens. Similar patterns of *C. trachomatis* genotypes were observed before and after MDA.

Combined ofr (*ompA* gene) and rg1 sequencing revealed that *C. trachomatis* ocular and urogenital genotypes grouped exclusively according to the anatomical site of collection. Similar *C. trachomatis* urogenital tract genotypes (D, E, G, J, and Ja) were detected in patient urine samples pre- and post-MDA and suggest that the overall *ompA* genotypic distribution remained relatively stable despite antibiotic treatment.

Typeable *C. trachomatis* isolates detected in ocular swabs were genetically homogeneous throughout the study population, except for 15 samples containing single-nucleotide variations. This aligns with findings reported in a previous study investigating conjunctival samples in Northern Territory, Australia (29). Additionally, there were no urogenital genotypes detected in ocular swabs. Together, this evidence indicates that urogenital strains of *C. trachomatis* are not making a material contribution to eye infections in Nauru. Across the surveys, there was an average 7.2% probability that participants within the same household were sampled in both surveys. The observed separation between types by site does not rule out the possibility that crossover can occur, as indeed has been reported from elsewhere in case (23), but rather that it is unlikely to be an important public health issue.

Our study genotyped large sample sets for two anatomical sites in the same small population with a high prevalence of both trachoma and urogenital tract infections, over a short investigative period. The extent of crossover is not well quantified; we hypothesize it would be more likely to be observed in settings where both types of infection are at a high prevalence. Earlier studies investigating urogenital types did not detect either trachoma genotypes in urogenital specimens or urogenital genotypes in eye swabs but were not performed in co-endemic populations (22, 29–31). It is certainly possible for crossover infections to occur, but in the absence of detectable evidence in a highly co-endemic population, we suggest that it is a rare event (23, 32–34). It is possible that the high prevalence of both urogenital and eye infection in this population reduces the likelihood of crossover: prolonged infection with *C. trachomatis* is followed by type-specific, partial immunity, and there is some evidence that cross-mucosal protection may be stimulated. The single detection of an LGV variant L1 from a urogenital tract specimen in our study highlights the need to assess the occurrence of the less common, more invasive lymphogranuloma venereum in this community.

We observed a high proportion of samples for which we could not produce PCR amplicons suitable for sequencing (202/406, 49.8%), so therefore it could not be typed. We focused on the *ompA* gene, even though as a single-copy gene, it may be more difficult to amplify than other *C. trachomatis* genes because the Sanger sequencing approach based on *ompA* provides the best resolution in distinguishing closely related genotypes, such as J and subvariant Ja, for instance. Low typeability of clinical specimens is a recurring challenge for characterizing the distribution of *C. trachomatis* genotypes at the population level. In our study, it most often occurred in urine and ocular specimens with high C_T_, indicative of the low load of *C. trachomatis*. Low bacterial load has been consistently reported as a barrier to investigating circulating genotypes from urine samples (29, 35, 36). High success rates in genotyping from urine specimens have been achieved by a nested PCR procedure (35) and with restriction fragment length polymorphism (RFLP) methods using nucleic acid extracted from urine pellets prepared from 5 mL of urine (36). This strategy was not able to detect mixed infections but allowed us to distinguish predominant genotypes in clinical specimens to achieve our goals of genetic surveillance. The likelihood of success using whole-genome sequencing approaches in the study was also limited, especially if samples are degraded or of low bacterial load (i.e., those that failed initial Sanger sequencing) (37). Future genomic studies could incorporate more comprehensive exploration of population-level genomics such as sensitive agnostic shotgun sequencing methodologies to improve strain detection over PCR and Sanger sequencing techniques.

To the best of our knowledge, this is the only typing study to describe the contemporaneous distribution of genotypes at ocular and urogenital anatomical sites in a population co-endemic for ocular and non-ocular *C. trachomatis* infections. Additionally, this is the only published study of *ompA* genotypes of *C. trachomatis* in Nauru and one of the largest in the Pacific region. There was no evidence of crossover infections between the two anatomical sites despite the high prevalence of both trachoma and urogenital infections in the population. It may be important to periodically survey chlamydial genetics as Nauru moves toward elimination of trachoma as a public health problem.

## Data Availability

De-identified participant data are available on reasonable request addressed to the corresponding author under certain conditions (with the consent of all participating centers and with a signed data access agreement). Novel ofr sequences were deposited into GenBank (accession numbers PX431604-PX431614).
